# Ammonium Glycyrrhizinate and Bergamot Essential Oil Co-Loaded Ultradeformable Nanocarriers: An Effective Natural Nanomedicine for In Vivo Anti-Inflammatory Topical Therapies

**DOI:** 10.3390/biomedicines10051039

**Published:** 2022-04-30

**Authors:** Maria Chiara Cristiano, Nicola d’Avanzo, Antonia Mancuso, Martine Tarsitano, Antonella Barone, Daniele Torella, Donatella Paolino, Massimo Fresta

**Affiliations:** 1 Department of Experimental and Clinical Medicine, University “Magna Græcia” of Catanzaro Campus Universitario-Germaneto, Viale Europa, 88100 Catanzaro, Italy; mchiara.cristiano@unicz.it (M.C.C.); antonia.mancuso@unicz.it (A.M.); barone@unicz.it (A.B.); dtorella@unicz.it (D.T.); 2 Department of Pharmacy, University “G. d’Annunzio” of Chieti-Pescara, Via dei Vestini n.31, 66100 Chieti, Italy; nicola.davanzo@unich.it; 3 Department of Health Science, University “Magna Græcia” of Catanzaro Campus Universitario-Germaneto, Viale Europa, 88100 Catanzaro, Italy; martine.tarsitano@studenti.unicz.it (M.T.); fresta@unicz.it (M.F.)

**Keywords:** ammonium glycyrrhizinate, bergamot essential oil, ultradeformable nanocarriers, multi-drug carriers, in vivo anti-inflammatory activity

## Abstract

Bergamot essential oil (BEO) and Ammonium glycyrrhizinate (AG), naturally derived compounds, have remarkable anti-inflammatory properties, thus making them suitable candidates for the treatment of skin disorders. Despite this, their inadequate physicochemical properties strongly compromise their topical application. Ultradeformable nanocarriers containing both BEO and AG were used to allow their passage through the skin, thus maximizing their therapeutic activity. Physicochemical characterization studies were performed using Zetasizer Nano ZS and Turbiscan Lab^®^. The dialysis method was used to investigate the release profile of the active compounds. In vivo studies were performed on human healthy volunteers through the X-Rite spectrophotometer. The nanosystems showed suitable features for topical cutaneous administration in terms of mean size, surface charge, size distribution, and long-term stability/storability. The co-delivery of BEO and AG in the deformable systems improved both the release profile kinetic of ammonium glycyrrhizinate and deformability properties of the resulting nanosystems. The topical cutaneous administration on human volunteers confirmed the efficacy of the nanosystems. In detail, BEO and AG-co-loaded ultradeformable vesicles showed a superior activity compared to that recorded from the ones containing AG as a single agent. These results are promising and strongly encourage a potential topical application of AG/BEO co-loaded nanocarriers for anti-inflammatory therapies.

## 1. Introduction

Essential oils and phytotherapic molecules overall caught the attention since ancient times [1,2,3,4], and in recent decades several research groups tried to exploit their benefits, improving their poor physicochemical properties using nanotechnologies [5,6,7]. In fact, nanotechnologies and drug delivery systems have already obtained plenty of experience behind them, thus allowing optimization of common therapies; and although few nanomedicines are actually on market, the advantages in their use remain undoubted [8].

**Figure 1:**
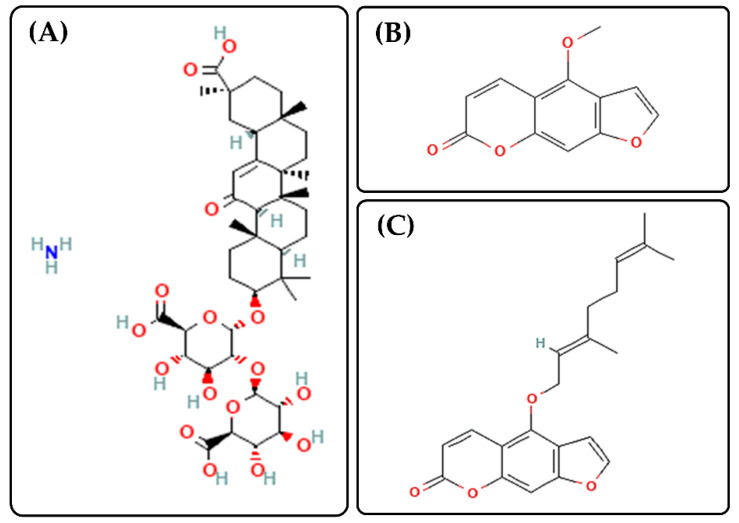
Chemical structures of ammonium glycyrrhizinate (AG, Panel (**A**), https://pubchem.ncbi.nlm.nih.gov/compound/62074#section=2D-Structure, accessed on 13 April 2022), Bergapten (Panel (**B**), https://pubchem.ncbi.nlm.nih.gov/compound/2355#section=2D-Structure, accessed on 13 April 2022) and Bergamottin (Panel (**C**), https://pubchem.ncbi.nlm.nih.gov/compound/5471349#section=2D-Structure, accessed on 13 April 2022).

Citrus bergamia, originally from the extreme south of the Italian peninsula, is known as Bergamot, and its essential oil extracted (BEO), is world-famous for its several properties and uses, such as antimicrobial and anti-inflammatory activity, hypolipidemic and hypoglycemic features, as well as for the food industry [9,10,11,12,13]. BEO is composed of a volatile fraction and a non-volatile one, the latter includes compounds, such as bergapten and bergamottin (Figure 1) [14]. Indeed, several studies investigated the role of bergapten in anti-inflammatory activity, showing its capability to reduce the levels of TNF-α and IL-6, also modifying the expression of COX-2 and i-NOS [15,16]. Moreover, bergapten, preventing the accumulation of ROS, acts as an antioxidant agent, fundamental in the inflammatory site [17].

Another Mediterranean species of pharmaceutical interest is glycyrrhiza glabra, especially glycyrrhizin acid and its derivatives, such as ammonium glycyrrhizinate (AG, Figure 1) [18,19]. In addition to BEO, AG acts at the level of COX-2, but also on prostaglandin E2 (PGE2) and nitric oxide, thus having an important role in the suppression of inflammatory processes too [19,20].

Despite the aforementioned remarkable anti-inflammatory properties of these two natural products, they showed a poor dermal and trans-dermal applicability in response to their unsuitable physicochemical features that limit their capability to cross the stratum corneum, as regards ammonium glycyrrhizinate, and/or because of their irritative/allergenic effects, as regards bergamot essential oil [9,21,22]. Based on this, the aim of this work was to exploit the anti-inflammatory synergic effects of both plant derivatives, encapsulating them into ultradeformable nanocarriers, to obtain a nano-formulation capable of contrasting inflammatory skin states efficiently. In fact, ultradeformable nanocarriers, thought for transdermal delivery of several compounds, can move through the skin, deforming themselves and at the same time keeping intact their conformation, thus preserving their cargos, and providing a suitable accumulation of them close to the inflammation sites [23,24]. Ultradeformable nanocarriers can be considered as an evolution of traditional liposomes. These systems are made up of phospholipids and edge activators, able to fluidify their lipid bilayer, increasing their deformability [25,26]. To date the exact mechanism of ultradeformable nanocarriers penetration through skin is not completely understood. In any case, several research groups demonstrated that they efficiently penetrate through the stratum corneum, when they are applied in non-occlusive condition, since the osmotic gradient moves their passage through skin [27,28].

In these attempts, the nanosystem was optimized focusing on the ability of BEO to improve both the physicochemical features and pharmacological effect of resulting therapeutic ultradeformable nanocarriers, when it was co-loaded with AG. These formulations were investigated in terms of average size, PDI, net surface charge, physical stability, entrapment efficiency capability of bioactives, in vitro kinetic release and storability, by optimizing the freeze-drying process. Moreover, the co-encapsulation of BEO and AG improved both the deformability index and in vivo anti-inflammatory properties of resulting nanovesicles compared to the ones containing only AG, showing, at the same time, well-accepted and safe profiles on healthy human volunteers.

Therefore, the obtained results strongly encourage a potential topical application of AG/BEO co-loaded ultradeformable nanocarriers to efficiently counter the skin inflammation states.

## 2. Materials and Methods

### 2.1. Materials

Phospholipon 90G^®^ (PL 90G), was provided by Lucas Meyer C. (Lucas Meyer Cosmetic, Hamburg, Germany). Ethanol, sodium cholate (SC), ammonium glycyrrhizinate (AG) were purchased from Sigma–Aldrich (Milan, Italy), while bergamot essential oil (BEO) was provided by H. Reynaud et Fils Matières Premieres Aromatiques (Saint-Didier, France). Cellulose membranes Spectra/Por MWCO 10000 Da were obtained from Spectrum Laboratories Inc. (Eindhoven, The Netherlands). All other chemicals used in the study were of analytical grade and no further purification was necessary.

### 2.2. Ultradeformable Nanocarriers Preparation and Purification

The ultradeformable nanocarriers were obtained according to the thin layer evaporation method as previously reported, with some modifications [29]. Briefly, PL90G^®^ (88 mg) and SC (12 mg) were dissolved in ethanol. A thin lipid film was obtained by removing the organic solvent using a Rotavapor^®^ (Büchi Italia, Milan, Italy) and, finally, hydrated using an hydroalcoholic solution (water:ethanol 93:7% *v*/*v*, 6 mL). The achieved vesicular suspension was extruded using polycarbonate porous filters (400, 200, and then 100 nm), under nitrogen flux, to reduce the average size and obtain a homogenous nanovesicles’ size distribution. The resulting colloidal suspensions were then kept at 4 °C up to the further investigations. Therapeutic ultradeformable nanocarriers were obtained by solubilizing AG, BEO, or both in the lipid mixture during the preparation phase. The exact compositions of formulated vesicular systems are reported in Table 1.

### 2.3. Physicochemical Characterization

All formulations were characterized in terms of mean size, size distribution and net surface charge, by dynamic light scattering (DLS) technique using a Zetasizer Nano ZS (Malvern Instruments Ltd., Worcestershire, UK).

Empty and drugs-loaded ultradeformable nanocarriers were also analyzed by the Turbiscan Lab^®^ Expert system to estimate their stability profiles, as previously described [30]. Variation of backscattering (ΔBS) and transmittance (ΔT) signals were measured as a function of sample height and the resulting data were used to predict the occurrence of potential destabilization phenomena (i.e., flocculation, sedimentation, and/or creaming) [30]. Furthermore, the global destabilization kinetic profile (TSI) was evaluated as a function of time. Experimental data were correlated in percentage to the light flux of two reference standards constituted by a polystyrene latex suspension (absence of transmission and maximum backscattering) and a silicon oil (maximum transmission and absence of backscattering).

### 2.4. High Performance Liquid Chromatography (HPLC) Instrument

The quantification of AG and BEO was carried out by using an HPLC (VarianInc., Palo Alto, CA, USA), equipped with a PDA MD-1510. A reverse phase C18 column (Zorbax Extend C18 column, size 250 mm × 4.6 mm, 5 μm) operating at room temperature, was used during the analysis for both drugs. AG was quantified as previously described by Barone et al. [31]. On the other hand, for BEO two of the main components of its non-volatile fraction, i.e., Bergapten and Bergamottin [14], were used for the essential oil quantification. Analyses were carried out at 319 nm and 310 nm, for the determination of bergapten and bergamottin, respectively. The mobile phase was composed of a mixture of water + 0.1% of formic acid(A)/methanol + 0.1% of formic acid(B). The samples were eluted in gradient condition: 0–1 min 40% of B, 1–4 min 90% of B, 4–8 min 90% of B, 8–9 min 99% of B, 9–17 min 99% of B, 17–18 min 40% of B, 18–30 min 40% of B. BEO was quantified using the external calibration curves of both bergapten and bergamottin.

The mobile phases were filtered through 0.2 μm polycarbonate filters prior to use and 20 μL of each sample was injected for the analysis. Data were acquired and processed by using Empower v.2 software (Water Spa, Milford, CT, USA).

### 2.5. Evaluation of Entrapment Efficiency of Natural Compounds

To quantify amounts of ammonium glycyrrhizinate and BEO entrapped in the ultradeformable nanocarriers, drug-loaded nanovesicles were placed in polycarbonate tubes and then centrifuge at 100,000× *g* at 4 °C for 1 h, through a Beckman Optima ultracentrifuge™ (Bechman Coulter Inc., Fullerton, CA, USA) equipped with a TL S55 fixed angle rotor. The obtained pellet was separated from the supernatant and solubilized in cold methanol. The entrapment efficiency was evaluated according to the Equation (1) following reported:

(1) $$E.E.\%=\left(\frac{Dp}{Dtot}\right)\times 100$$

where *Dtot* was the total amount of bioactive compound used during the ultradeformable nanocarriers preparation and *Dp* was the amount of compound quantified in the pellet. Empty nanovesicles with the same lipid composition were used as blank.

### 2.6. Freeze-Drying Stability Studies

Then, 500 microliters of each formulation were lyophilized by using a freeze-drying apparatus (VirTis SP scientific sentry 2.0; SP Industries, Warminster, PA, USA) equipped with a vacuum pump (B14 model; Carpanelli S.p.a., Bologna, Italy). The samples were placed in the suitable tubes with different cryoprotectants at two different concentrations (2% and 5% *w*/*v*) and frozen in liquid nitrogen. Then, the frozen samples were transferred in the freeze-drying chamber and cryo-dried for 24 h. The obtained lyophilized powers were maintained at room temperature for 5 days, and then rehydrated by slight shaking. The physicochemical properties of resulting suspension were evaluated by DLS.

### 2.7. In Vitro Ammonium Glycyrrhizinate Release Kinetic

AG release profiles from vesicles were obtained according to the dialysis method previously reported, with some modifications [32]. A hydroalcoholic solution (H2O:Ethanol 70:30, *v*/*v*) was used as a receiver compartment based on amphiphilic features of AG maintained at 32 °C ± 0.5 °C [33] throughout the entire analysis. At pre-determined time intervals (0, 30 min, 1, 2, 4, 6, 8, 24, 48, 72, 96, 120, 144, 168, 192, 216 h), 1 mL of release medium was withdrawn and replaced with the same volume of fresh one. Collected samples were overnight dried in a Savant SpeedVac concentrator (Thermo Fisher Scientific, Monza, Italy) and then re-solubilized with fresh methanol. No interference peaks were observed. The percentage of drug release (*D. R. %*) was calculated according to Equation (2):

(2) $$D.R.\;\left(\%\right)=\left(\frac{Dr}{Dl}\right)$$

where *Dr* is the amount of released AG at the time t and *Dl* is the amount of entrapped drug. An empty formulation under the same conditions was used throughout the experiment as blank.

### 2.8. Deformability Assay

The Deformability Index (*D.I*.) of AG-, BEO- and MD-loaded ultradeformable nanocarriers was determined, as described in our previous study [34]. Briefly, vesicles were analyzed in terms of mean size before and after their extrusion through polycarbonate filters (with a proper pore size). In detail, the pore size of the filter was chosen based on the initial average size of nanovesicles (one third of it). The extrusion was carried out maintaining a constant pressure equal to 5 bar for 10 min. The deformability properties, reported as Deformability Index (*D.I.*), were determined according to the following Equation (3):

(3) $$D.I.=J\left(\frac{d0}{p}\right)x\left(\frac{d0}{d0-d1}\right)$$

where *J* is formulation’s fraction recovered after extrusion process (values between 0 and 1), *p* denotes pores dimension, *d*0 and *d*1 are ultradeformable nanocarriers mean diameters, before and after extrusion, respectively.

### 2.9. In Vivo Evaluation of Topical Tolerability

The in vivo tolerability of empty and loaded ultradeformable nanocarriers was evaluated in 10 healthy human volunteers by using the non-invasive reflectance spectrophotometer X-Rite SP60 (X-Rite Incorporated, Grandville, Michigan, MI, USA), having 0° illumination and 45° viewing angles and calibrated with a white standard traceable to the National Bureau of Standard’s perfect white diffuser [31]. The instrument was able to detect any change in the skin color following the application of formulations. Before starting the in vivo study, the volunteers (both sexes and with a mean age of 30 ± 5 years) were informed on the study and they provided their written and informed consent; moreover, they were left to acclimate for 20 min at room conditions (25 ± 1 °C; 40–50% humidity). In detail, several skin sites (1 cm^2^ in diameter and at least 2 cm of distance between sites to avoid contamination) on the ventral surface of the foreman of each volunteer were demarcated and analyzed to obtain baseline values before the treatments. Then, 200 µL of formulation or of saline solution (used as negative control, NaCl 0.9% *w*/*v*) was applied on the respective and chosen skin sites. After treatment, and at a prefixed time, the treated sites were cleaned to remove any traces of formulations and then erythema index (*E.I*.) values were monitored and recorded, according to the following equation:

(4) $$E.I.=100\left[log\frac{1}{R560}+1.5\left(log\frac{1}{R540}+log\frac{1}{R580}\right)-2\left(log\frac{1}{R510}+log\frac{1}{R610}\right)\right]$$

where *R* was the reflectance at different wavelengths (510 nm, 540 nm, 560 nm, 580 nm, and 610 nm). The reported results were obtained subtracting the baseline values from the values of *E.I*. after formulations’ application, as a function of the time.

### 2.10. In Vivo Anti-Inflammatory Evaluation of Vesicles

The method previously described was used also to evaluate the efficacy of loaded ultradeformable nanocarriers in terms of anti-inflammatory activity in comparison with hydroalcoholic solution of ammonium glycyrrhizinate. The formulations were tested on 10 informed healthy human volunteers by using reflectance visible spectrophotometer, SP60 (X-Rite Incorporated, Grandville, Michigan, MI, USA). To mimic an inflammation of the skin, 200 µL of methylnicotinate solution (0.2% *w*/*v*) were applied on demarcated skin sites. After induced inflammation, the same skin sites were treated with 200 µL of AG solution, Formulation A, Formulation C, Formulation G, and saline solution as negative control. Analyses was performed at 30 min, 1 h, 2 h, 4 h, 6 h, and 8 h after treatment. Results are reported as differences between the *E.I.* values measured at different time points (Equation (4)) and *E.I*. value of baseline ± standard deviation.

All studies involving human subjects were carried out in accordance with the Declaration of Helsinki guidelines. The protocols were approved by the Research Ethics Committee of the “Magna Graecia” University of Catanzaro (Ethics approval numbers: 390/2019; 392/2019).

### 2.11. Statistical Analysis

Statistical analysis was carried out with the ANOVA test. Bonferroni t-test was used for comparison between samples. The significance levels were set at * *p* < 0.05 and ** *p* < 0.001. Results are expressed as the mean ± standard deviation (S.D.).

## 3. Results and Discussion

### 3.1. Physicochemical and Technological Characterization of Ultradeformable Nanocarriers

Physicochemical parameters of drug delivery systems play a pivotal role during the penetration process through the skin [35]. In these attempts, the mean size, polydispersity index, and net surface charge values of all realized formulations were measured, to evaluate their potential therapeutic applicability and the impact of the encapsulated compound(s) on these features (Table 2). All analyzed formulations showed an average size below 140 nm, with a slight variation in response to the encapsulation of AG and BEO, alone and in combination. In detail, the empty formulation (formulation A) was characterized by a mean size equal to 135 ± 2 nm and a polydispersity index of 0.109 ± 0.019, thus showing a homogeneous size distribution. Adding AG to the formulation, a proportional mean size reduction with respect to the increased amount of encapsulated compound, was recorded (Table 2). Indeed, the formulation D, prepared using the highest AG concentration, showed a mean size value equal to 105 ± 1 nm, statistically smaller than formulation A (*p* < 0.001). It is possible to suppose that AG, thanks to its amphiphilic nature, could act as an anchor interposed between the lipid bilayer and the aqueous core, thus causing a reduction in the vesicle diameter as a consequence of a structural rearrangement. A similar trend was also confirmed in formulations F, G, and H, prepared co-delivering AG and BEO, despite the presence of BEO reducing the shrinkage induced by AG encapsulation. In any case, the mean size of both empty and drug(s)-loaded ultradeformable nanocarriers suggested an acceptable value for a potential topical application and for the delivery of payloads through skin [36,37]. Moreover, the encapsulation of bioactive compounds did not compromise the suitable size distribution of resulting nanovesicles, recording a PdI value below 0.2 for all formulations, thus showing a narrow size distribution [29].

Another investigated parameter was the net surface charge, recording values below −30 mV for all analyzed samples (Table 2). The strongly negative values of net surface charge suggest a good stability over time of the vesicles, thanks to the electrostatic repulsion that arises between nanosystems, thus preventing potential instability phenomena, such as aggregation, agglomeration, and/or flocculation [38,39]. Significant variations of this parameter (more negative values) were obtained for formulations G and H, containing BEO and AG at the highest amount. Probably, this was due to the occurred interaction between AG and BEO components, thus leading to the exposition of different negatively charged groups. These results emphasized a potential further stabilization of resulting colloidal nanosystems by the co-delivery of both bioactive compounds.

**Figure 2:**
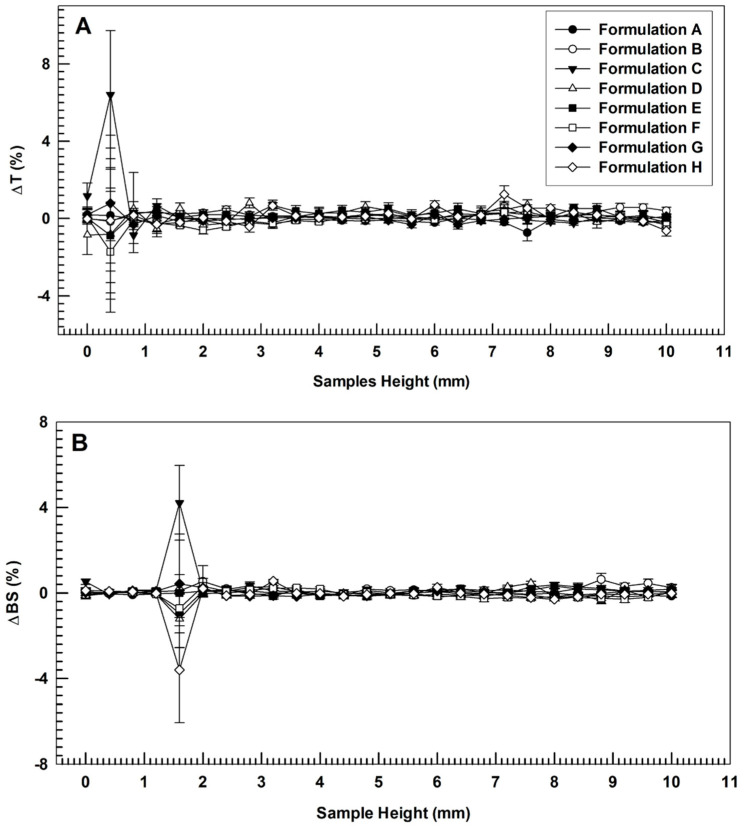
Variation of transmission (Panel (**A**)) and backscattering (Panel (**B**)) profiles of empty (A), AG-loaded ultradeformable nanocarriers (B–D), BEO-loaded ultradeformable nanocarriers (E) and multi-drug ultradeformable nanocarriers (F–H). The analysis was performed at 25 °C and data are representative of five independent experiments. Results are reported as a function of sample height (mm) and time (0–60 min).

To confirm physical stability of nanosystems deduced by net surface charge evaluation, the formulations were analyzed by the Turbiscan Lab^®^ Expert instrument. This last allows to predict potential destabilization phenomena of the colloidal suspension without altering the sample, through the measurement of transmission and backscattering profiles as a function of high amounts of sample and time [30]. The stability profiles of investigated formulations were shown in Figure 2.

As shown in Figure 2, no significant modification of Turbiscan backscattering profiles of colloidal suspensions occurred when different amounts AG (alone or in combination with BEO) were used to prepare ultradeformable nanocarriers (overlapped curves). No coalescence, sedimentation, flocculation, or clarification phenomena were detected, as demonstrated by ΔT and ΔBS profiles that remained in a narrow range (±5) during the entire analysis [40]. The only signs of profiles alteration were recorded at specific sample heights (up to 2 mm). However, as previously reported, these variations are not related to instability phenomena but were due to air inclusion at the bottom of the vial [41].

**Figure 3:**
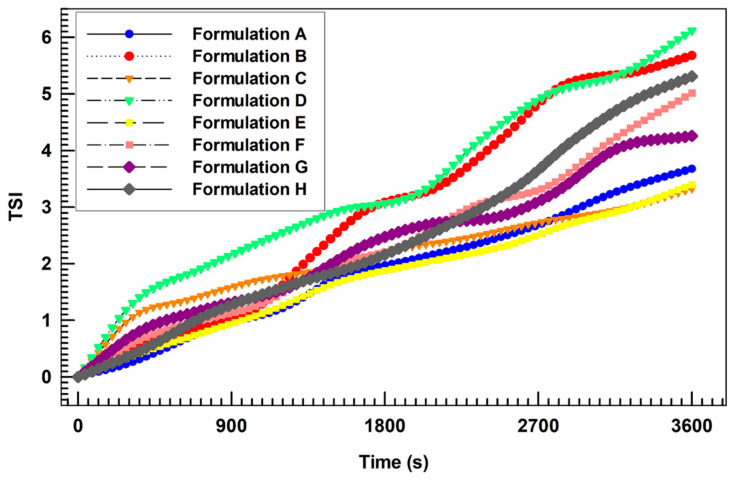
Turbiscan Stability Index (TSI) profiles of all prepared formulations, obtained by using the Turbiscan Lab ^®^ Expert.

The long-term stability of investigated formulations was further confirmed by the analysis of destabilization kinetic analysis in terms of TSI (Turbiscan Stability Index). Indeed, as shown in Figure 3, the curves obtained for different formulations demonstrated very similar profiles without significant differences among each other. In particular, the formulation D (containing only AG at the highest amount) demonstrated the highest destabilization profile. Conversely, it is noteworthy that the formulation H (containing both BEO and AG at the same amount of formulation D) showed a more stable profile, suggesting a potential (despite weak and no-significant) stabilization of AG-loaded nanovesicles in response to the co-loading of BEO. It is possible to assume that BEO, acting as a lipid bilayer fluidizing agent (as demonstrated in Section 3.3.), restored a suitable membrane flexibility lost in response to the encapsulation of a high amount of AG (also demonstrated in Section 3.3.). The result of this action was the improvement of the physical stability of resulting multi-drug ultradeformable nanocarriers.

Despite the demonstrated physical stability, the storability of aqueous colloidal suspension remains one of the main challenges for the translation of nanomedicine from bench to bedside. In fact, the massive amount of water in these formulations can compromise the proper storage of nanosystems, by promoting both chemical instability (e.g., hydrolysis processes) and/or the microbial growth [42,43]. To overcome these drawbacks, the applicability of the freeze-drying process was investigated during the study. In particular, several cryoprotectant agents were investigated (at two different concentrations, i.e., 2% and 5% *w*/*v*) and the physicochemical parameters of both empty and therapeutic ultradeformable nanocarriers were evaluated after lyophilization.

In Table S1 (Supplementary Materials), mean size and PdI values are reported for each formulation and for every amount of used cryoprotectants. As shown, after the freeze-drying process, the mean size values of vesicular systems underwent an increase during re-hydration step. Probably this phenomenon was due to the presence of small amounts of ethanol [44] (due to the buffer used during hydration phase, i.e., water:ethanol 93:7 *v*/*v*) and the concomitant absence of cholesterol [45] (well-known bilayer packing agent), thus leading to a high permeability of phospholipid bilayer and then the potential insertion of sugar molecules into it [46]. This increase was less evident in formulations with the highest amount of AG (5 and 7.5 mg mL^−1^) both without or with BEO, i.e., the samples C, D and G, H, respectively. Based on the high phospholipid bilayer permeability hypothesized above and the amphiphilic properties of AG [47], we can speculate that the proportional improvement of nanovesicles’ stability to the freeze-drying process in response to the increase in AG amount, was due to the physical localization of this drug. AG molecules may potentially interact with phospholipids’ hydrophobic chains with its corticosteroid similar ring, while hydrophilic groups should be located toward the aqueous compartments, thus providing a potential interaction with hydrophilic head of phospholipids and enhancing the bilayer stability. Based on these hypotheses, AG could play a cholesterol-like function when it is encapsulated in a suitable amount. Moreover, despite the undoubted increase, the average sizes of formulation C, D, G, and H was always below 400 nm, regardless of the type and percentage of cryoprotectant used, except for formulations C and D lyophilized by using mannitol at 5% *w*/*v*. These results suggested the suitable application of freeze-dried process to the formulation C, D, G and H, that showed proper physicochemical properties after re-hydration, suggesting a not compromised transdermal permeation [36,37]. In particular, the best results were obtained when mannose for formulation C, sucrose for formulation D, and mannitol for formulations G and H, were used as cryoprotectants. These findings highlight the importance of investigating the proper cryoprotectant and then optimizing its amount for each formulation to ensure a suitable freeze-drying process [48].

Conversely, the not cited formulations were found to be excessively susceptible to the process, with an unacceptable increase in the mean size, demonstrating that they were unsuitable for post-freeze-drying re-suspension in some cases.

### 3.2. Entrapment Efficiency Evaluation

The entrapment efficiency is a key parameter that shows the ability of a nanosystem to embed a certain amount of payload(s). In these attempts, its evaluation is crucial during the early stage of nanomedicine realization, thus becoming pivotal to predict its potential therapeutic application and in vivo behavior [49].

In Table 3, the encapsulated amounts of AG and BEO in all formulations, both alone and in combination, are reported. About AG, the data show that the ability of ultradeformable nanocarriers to contain the licorice extract is dependent on the added amount during preparation. In detail, the nanovesicles’ entrapment capability and drug loading content, increased by improving the drug amount added during the realization phases [50], leading to an E.E. greater than 70% for the two formulations containing the highest AG concentrations (formulation C and D). Probably, due to its amphiphilic features, ammonium glycyrrhizinate was able to find space both in the aqueous compartments of the ultradeformable nanocarriers and in the lipidic districts arranging itself with its hydrophilic and anionic groups and its corticosteroid similar structure [47], respectively. A similar trend was shown also when AG was added in combination with BEO, highlighting that the presence of essential oil did not prevent the suitable encapsulation of AG, despite a slight reduction in its entrapment efficiency was demonstrated for formulation G compared to formulation C. Regarding bergamot essential oil, the entrapment efficiency studies were carried out evaluating two of the main components of its non-volatile fraction, i.e., bergamottin and bergapten, through the use of the proper calibration curves, as described above in Section 2.5.

Conversely, as reported in Table 3, the entrapment efficiency of BEO was significantly affected by the co-loading of AG. Namely, the entrapped amount of BEO underwent a progressive reduction proportionally to the AG concentration increase. Probably, this was due to the physical localization of high amounts of lipophilic AG residuals in the bilayer structure, thus resulting in a steric hindrance that could justify the reduced embedding of BEO.

### 3.3. In Vitro Ammonium Glycyrrhizinate Release Studies

**Figure 4:**
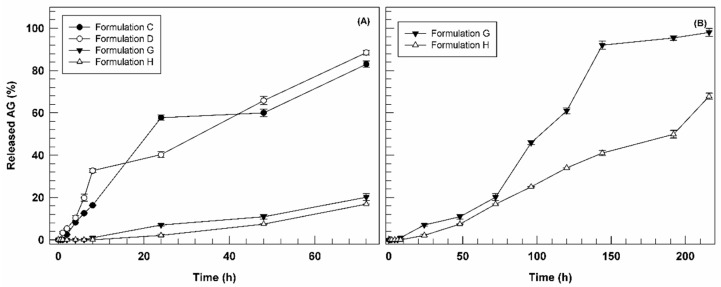
Release profile of AG-loaded ultradeformable nanocarriers as a function of time (Panel (**A**): 0–72 h; Panel (**B**): 0–216 h). Results are the average of five independent experiments ± standard deviation.

Based on entrapment efficiency studies, the in vitro AG release profiles kinetics of formulations C, D, G, and H were analyzed as a function of incubation time (Figure 4).

In Figure 4, we reported the release profiles as a function of short incubation time (72 h) for all investigated formulations (panel A) and long one (7 days) for formulations G and H (panel B). In fact, observing the figure, it is possible to note immediately the different behavior between AG-loaded formulations (C and D) and the multi-drug formulations (G and H) in terms of release profiles. Nanovesicles containing only AG demonstrated a biphasic kinetic, releasing ca. 60 and 40% of loaded bioactive after 24 h, for formulation C and D, respectively, and then reaching 80% of AG cumulative release after 72 h of incubation (Figure 4A). Conversely, the multi-drug formulations G and H showed a marked reduction in release kinetics at 72 h of incubation, reaching only 20.15 ± 1.65% and 16.90 ± 0.2%, respectively. Since the only difference between the two pools of formulations is the presence of BEO in formulation G and H, we can suppose that their retarded release could be associated with essential oil and/or at one or more its components. In fact, due to the lipophilic nature of BEO, its components could be interposed between phospholipids of ultradeformable nanocarriers, leading to a potential chemical interaction with the lipophilic portion of AG, thus delaying the release of this last. Based on the release data obtained in the first 72 h, we decided to monitor the release profiles of formulations G and H until 7 days (216 h). As shown in Figure 4B, only after 120 h formulation G released more than 50% of entrapped AG, reaching almost 100% in ca. 150 h. On the other hand, formulation H showed an even more delayed AG release (ca. 65% after 7 days). These results support our hypothesis, confirming the ability of BEO to retain AG in the ultradeformable nanocarriers structure. It is also possible that the main release of entrapped compound become more evident for formulations G and H after long incubation time due to the destabilization effect of ethanol contained in the receiver compartment (30% *v*/*v*) on nanovesicles ultrastructure after suitable incubation time.

The obtained release data can be considered as promising results because the combination of two natural compounds, AG and BEO, seems to allow a more controlled and sustained release of an anti-inflammatory bioactive compound. Based on this trend it is possible to suppose a potential reduction in the topical applications frequency, thus improving the patient compliance in a potential clinical application. Moreover, the slow-release rate found in vitro does not compromise the potential in vivo therapeutic activity of payloads. Indeed, it is possible to suppose that therapeutic nanovesicles can reach the inflammation site intact thanks to their deformability properties and then massively release the cargos in response to the physiopathology stimuli (e.g., warm, enzymatic activity, oxidative environment, etc.).

### 3.4. Deformability Index

Ammonium glycyrrhizinate is an amphiphilic compound [47] composed by a hydrophobic portion that potentially interacts with lipid structures of ultradeformable nanocarriers and by a hydrophilic portion that is probably exposed towards aqueous compartments. On the contrary, the high lipophilicity of BEO and its main components suggest its localization between lipids of nanocarriers’ bilayer.

**Figure 5:**
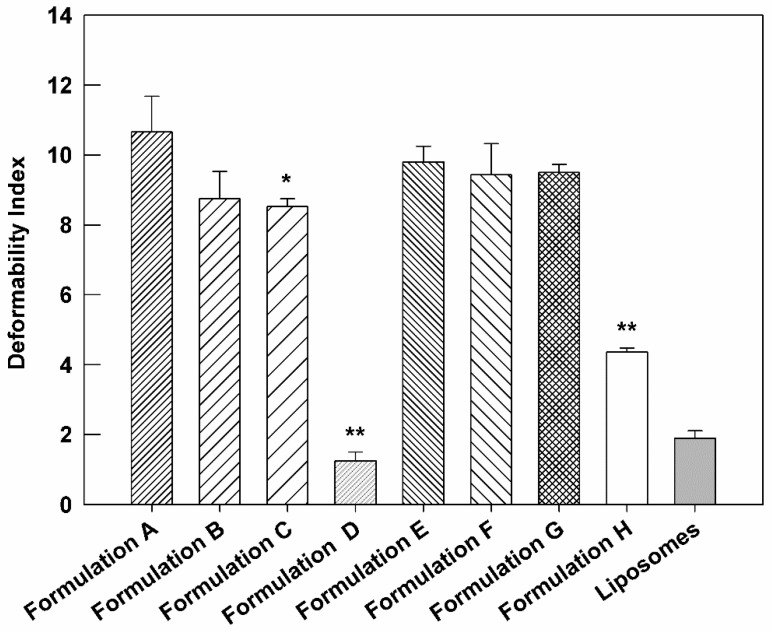
Deformability Index of compound(s)-loaded ultradeformable nanocarriers formulations, compared to empty ultradeformable nanocarriers (Formulation A) and liposomes, used as negative control. The results are expressed as the mean value of five independent experiments ± standard deviation. * *p* < 0.05 and ** *p* < 0.001 of formulation vs. Formulation A.

In these attempts, in order to confirm the preservation of suitable deformability of resulting therapeutic nanovesicles, this parameter was investigated for cargo-loaded ultradeformable nanocarriers and then compared to the empty one. Indeed, one of the main properties of ultradeformable nanocarriers is their high deformability value, which, in association with the small dimensions, makes them suitable for the transcutaneous application and, therefore, for the delivery of bioactive(s) through the stratum corneum of the skin. Figure 5 reports the deformability index of empty, AG-loaded, and AG-BEO co-loaded formulations. The obtained results were also compared with liposomes, used as a negative control, due to their distinct rigidity [51]. The empty formulation confirmed the high deformability index (*D.I.* = 10.66 ± 1.02). The addition of AG in ultradeformable nanocarriers reduced *D.I.* as a function of used compound concentration, and, in particular, this value strongly decreased in formulation D (*D.I.* = 1.25 ± 0.25), showing a similar value of liposomes (*D.I.* = 1.90 ± 0.21). These findings further confirm our hypothesis concerning the ability of AG to increase the bilayer rigidity at a high concentration (see Section 3.1). It is noteworthy that all multi-drug formulations showed a higher *D.I.* compared to the formulation containing only AG at the same amount, suggesting that the presence of BEO mitigates the stiffening effect induced by the AG on nanovesicles’ bilayer. In particular, the fluidizing effect of BEO was more evident in formulation H, showing a *D.I.* (4.36 ± 0.12) almost four times higher than formulation D (*D.I.* = 1.25 ± 0.25), which had the same amount of entrapped AG. Based on that, we can assume that BEO was useful to preserve proper deformability characteristics of ultradeformable nanocarriers, even in the presence of high AG concentrations.

### 3.5. In Vivo Studies on Human Health Volunteers

**Figure 6:**
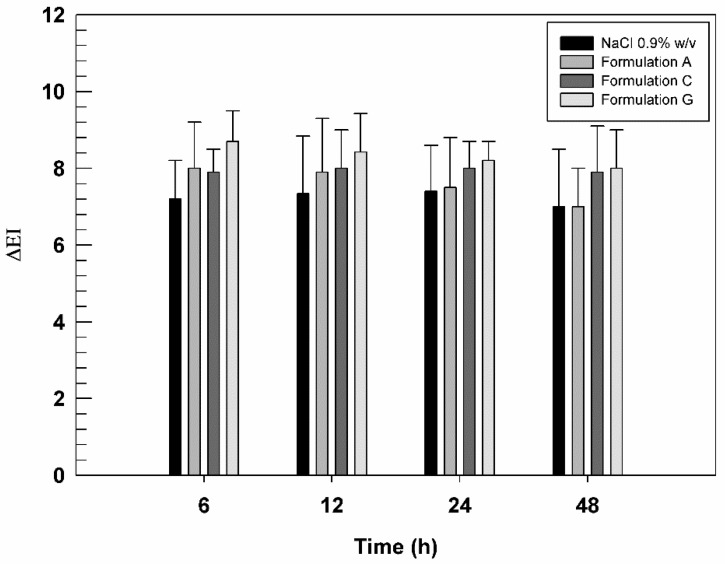
In vivo tolerability of saline solution (NaCl 0.9% *w*/*v*, used as negative control), Formulation A, Formulation C, and Formulation G, evaluated on human healthy volunteers and by using X-Rite apparatus. The results are expressed as mean values of erythematous index variation versus time. The results obtained for tested formulation are not statistically significant when compared with saline solution.

Based on deformability studies, Formulation C and G were considered the best formulations to test on human healthy volunteers. In fact, the chosen formulations contained the highest AG concentration that did not compromise the deformable properties of ultradeformable nanocarriers. Formulation A was used to evaluate the effect of empty nanovesicles. Their skin compatibility was tested on human healthy volunteers, measuring the variation of erythema index (ΔEI) after their application on the skin by using the non-invasive method of reflectance spectrophotometry. As reported in Figure 6, the three tested formulations were well-tolerated by the skin on volunteers, without significant differences in ΔEI value compared to saline solution, used as negative control.

In detail, no-occlusive conditions were maintained throughout the analysis because they are fundamental to allow the passage of ultradeformable nanocarriers through stratum corneum, following the aqueous osmotic gradient existing between external and internal layers of the skin [29].

Moreover, ΔEI values of Formulation G were slightly higher than other formulations, although these differences were statistically insignificant. Probably, some components contained in BEO induced a very slight irritation [52] at the application site while not altering the state of health of the skin and volunteer’s compliance.

Based on the obtained results and the high skin compatibility of tested formulations, their anti-inflammatory features were also investigated. In particular, the investigation was carried out on human healthy volunteers in order to evaluate the anti-inflammatory activity of AG-loaded nanovesicles (both as a single agent or in association with BEO) compared to AG hydroalcoholic solution. For this scope, it was necessary to induce a chemical inflammation on the skin of enrolled human subjects. At this purpose, the specific skin site was pre-treated with methyl nicotinate 0.2% *w*/*v* solution, thus leading to an induced erythema that was monitored during time.

Subsequently, saline solution, AG hydroalcoholic solution, formulations C and G were applied on respective skin site and the regression of erythema was measured and correlated with the pharmacological effects of samples.

**Figure 7:**
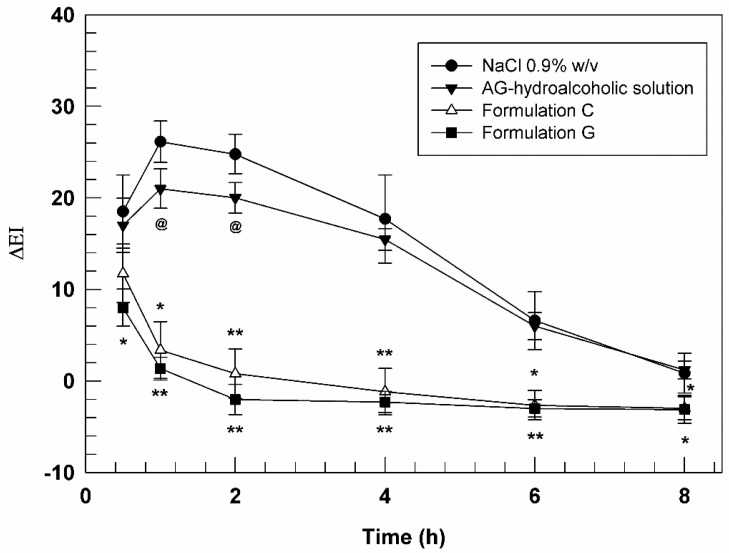
In vivo anti-inflammatory activity evaluation of AG hydroalcoholic solution, Formulation C and Formulation G. The results (mean values of five different experiments ± standard deviation) are expressed as erythema index variation (ΔEI) versus time. The AG-hydroalcoholic solution was compared to saline solution (@ *p* < 0.05), while data obtained testing formulation C and G were compared to the ones carried out by AG-hydroalcoholic solution. * *p* < 0.05, ** *p* < 0.001.

Figure 7 reported the curves of ΔEI as a function of time (30 min, 1, 2, 4, 6, and 8 h). In this graph, it is possible to note that the saline solution curve followed the trend of erythema which increased during the early hours and then decreased during experiment, indicating the physiological restoration of skin integrity. As expected, AG in free form showed a very weak efficacy in reducing chemically induced inflammation probably due to its poor ability to cross the stratum corneum. In fact, a slight reduction in ΔEI values (*p* < 0.05) induced by AG-hydroalcoholic solution compared to NaCl solution, was observed only at 1 and 2 h after application.

Formulation A, consisting in empty ultradeformable nanocarriers, did not demonstrate significant variations of chemically induced erythema, thus confirming the inertia of the empty vesicles and their inability to exercise a pharmacological activity per se (data not shown). On the other hand, the ultradeformable vesicles were demonstrated to significantly improve the pharmacological activity of loaded AG, allowing the passage of the drug through stratum corneum. In fact, both formulations C and G, containing an AG concentration of 3.9 ± 0.04 and 3.2 ± 0.064 mg mL^−1^, respectively, induced a significant decrease in erythema index from the early stages of analysis. In detail, Formulation C was able to reduce erythema index of ~6-folds after 1 h and even of ~25-forlds after 2 h of treatments, compared to AG-hydroalcoholic solution, confirming again the effectiveness of deformable vesicular systems to allow the passage of active ingredients [53] with unfavorable physicochemical properties through the skin.

A further improved effect was obtained using sample G. In fact, this formulation was able to further reduce the induced skin inflammation in comparison to formulation C, despite the lower amount of delivered AG. In particular, the differences between the formulation C and G were more relevant during the early stages of experiments, showing a statistical difference after 30 min and 1 h.

Based on the obtained results, BEO seemed to be able to enhance the anti-inflammatory activity of AG, providing a potential synergistic effect due to its well-known anti-inflammatory properties [14] and its capability to attenuate the stiffening effect provided by AG on bilayer structure. These results strongly support our hypothesis regarding the ability of BEO to improve the deformability of AG-loaded ultradeformable nanocarriers, thus allowing a more effective and a greater accumulation of payloads in the dermis, where AG can exert its effects.

## 4. Conclusions

In this study, we demonstrated the improved anti-inflammatory properties of ammonium glycyrrhizinate and bergamot essential oil, co-delivering these bioactives by ultradeformable nanocarriers.

In particular, the co-encapsulation of BEO into AG-loaded nanovesicles enhanced both the physicochemical properties of resulting formulation and its pharmacological effect. In fact, one of the main properties required for nanosystems with a dermal and/or transdermal application is to have proper deformability to cross intact the stratum corneum.

Unfortunately, this parameter was reduced when a high amount of AG (as a single agent) was encapsulated. Conversely, the co-encapsulation of BEO led to a partial restoration of deformability thus allowing a suitable topical application. Among different investigated formulations, the one that showed the best results was Formulation G (containing 3.2 ± 0.064 mg mL^−1^ and 2.6±0.034 mg mL^−1^ of AG and BEO, respectively). Indeed, this multi-drug nanovesicles demonstrated reasonable physicochemical properties, i.e., an average size below 130 nm, a narrow size distribution (PDI equal 0.120 ± 0.024), and a strong negative surface charge (−43.2 ± 1.6) that resulted in a high physical stability of colloidal nanosystem.

Moreover, BEO heavily affected the release profile kinetic of AG, thus providing a more controlled and sustained release up to 7 days, suggesting a reduction in application frequency in a potential therapeutic application and/or a reduced drug leakage during the crossing of corneous layer.

This effect did not compromise the in vivo therapeutic activity of this formulation, that in response to the higher deformability compared to nanovesicles with a similar amount of AG as single agent (8.52 ± 0.23 vs. 9.5 ± 0.25, respectively) was able to better delivery the bioactive molecules in the inflamed sites, maximizing the anti-inflammatory effect of payloads.

These promising results, in addition to the nanovesicles stability during the freeze-drying process, demonstrated the long-term stability/storability and the high efficiency of aforementioned formulation, thus emphasizing its potential topical anti-inflammatory application.

## Figures and Tables

**Table 1:** Composition of ultradeformable nanocarriers formulation.

Sample	AG (mg/mL)	BEO (% *w*/*v*)
A	-	-
B	3	-
C	5	-
D	7.5	-
E	-	1
F	3	1
G	5	1
H	7.5	1

**Table 2:** Physicochemical parameters of ultradeformable nanocarriers formulation.

Sample	Mean Size (nm)	PdI ^a^	Net Surface Charge (mV)
A	135 ± 2	0.109 ± 0.019	−35.9 ± 0.2
B	134 ± 1	0.110 ± 0.001	−32.6 ± 0.4
C	119 ± 1	0.117 ± 0.008	−37.3 ± 1.3
D	105 ± 1	0.168 ± 0.003	−39.0 ± 1.3
E	133 ±1	0.082 ± 0.026	−30.5 ± 0.8
F	126 ± 1	0.091 ± 0.007	−32.8 ± 0.6
G	129 ± 1	0.120 ± 0.024	−43.2 ± 1.6
H	124 ± 1	0.139 ± 0.022	−46.7 ± 0.4

^a^ PdI = Polydispersity Index

**Table 3:** Entrapment efficiency (E.E.) of the different vesicular formulations.

Sample	AG E.E. (%)	BEO E.E. (%)
B	39.5 ± 0.6	-
C	77.8 ± 1.0	-
D	75.1 ± 1.8	-
E	-	33.1 ± 0.2
F	45.2 ± 0.6	28.1 ± 0.7
G	65.0 ± 2.0	26.4 ± 1.3
H	74.2 ± 1.0	24.0 ± 0.9

## Data Availability

Data is contained within the article and Supplementary Files.

## Supplementary Materials

The following supporting information can be downloaded at: https://www.mdpi.com/article/10.3390/biomedicines10051039/s1, Table S1: Mean sizes and PdI values of formulations after freeze-drying.
