# Supplementary material for: Ammonium Glycyrrhizinate and Bergamot Essential Oil Co-Loaded Ultradeformable Nanocarriers: An Effective Natural Nanomedicine for In Vivo Anti-Inflammatory Topical Therapies
Source: Biomedicines. 2022 Apr 30;10(5):1039. doi: 10.3390/biomedicines10051039 (PMC9138283; doi:10.3390/biomedicines10051039)
Supplement: Supplementary file 1 [file biomedicines-10-01039-s001.zip › biomedicines-1705608-supplementary.pdf]

Supplementary Materials

Ammonium glycyrrhizinate and Bergamot essential oil co-loaded ultradeformable nanocarriers: an effective natural nanomedicine for in vivo anti-inflammatory topical therapies

Maria Chiara Cristiano [1*], Nicola d'Avanzo [2*], Antonia Mancuso [1], Martine Tarsitano [3], Antonella Barone [1], Daniele Torella[1], Massimo Fresta [3], Donatella Paolino [1*]

[1] Department of Experimental and Clinical Medicine, University "Magna Græcia" of Catanzaro Campus Universitario-Germaneto, Viale Europa, Catanzaro, 88100, Italy; mchiara.cristiano@unicz.it; antonia.mancuso@unicz.it; barone@unicz.it; dtorella@unicz.it; paolino@unicz.it

[2] Department of Pharmacy, University "G. d'Annunzio" of Chieti-Pescara, Via dei Vestini n.31, Chieti, 66100, Italy; nicola.davanzo@unich.it

[3] Department of Health Science, University "Magna Græcia" of Catanzaro Campus Universitario-Germaneto, Viale Europa, Catanzaro, 88100, Italy; martine.tarsitano@studenti.unicz.it; fresta@unicz.it

* Correspondence: paolino@unicz.it; Tel.: +39 09613694211

† These authors equally contributed

Table S1. Mean sizes and PdI values of formulations after freeze-drying.

Samples	Cryoprotectan (%w/v)		Mean size (nm)	PdI
A1	Sucrose	2%	351 ± 15	0.541 ± 0.050
A2		5%	285 ± 4	0.425 ± 0.042
A3	Trehalose	2%	389 ± 7	0.501 ± 0.069
A4		5%	401 ± 11	0.684 ± 0.023
A5	Mannose	2%	502 ± 14	0.537 ± 0.061
A6		5%	423 ± 9	0.498 ± 0.058
A7	Mannitol	2%	394 ± 5	0.608 ± 0.058
A8		5%	Unsuitable for resuspension	
B1	Sucrose	2%	616 ± 19	0.517 ± 0.060
B2		5%	447 ± 5	0.512 ± 0.028
B3	Trehalose	2%	535 ± 9	0.559 ± 0.028
B4		5%	588 ± 11	0.892 ± 0.064
B5	Mannose	2%	580 ± 69	0.705 ± 0.279
B6		5%	446 ± 6	0.580 ± 0.040
B7	Mannitol	2%	609 ± 22	0.554 ± 0.017
B8		5%	Unsuitable for resuspension	
C1	Sucrose	2%	278 ± 15	0.576 ± 0.130
C2		5%	414 ± 13	0.434 ± 0.081
C3	Trehalose	2%	281 ± 1	0.564 ± 0.007
C4		5%	340 ± 8	0.508 ± 0.024
C5	Mannose	2%	208 ± 5	0.512 ± 0.023
C6		5%	200 ± 1	0.439 ± 0.010

C7	Mannitol	2%	269 ± 23	0.694 ± 0.202
C8		5%	Unsuitable resuspension	
D1	Sucrose	2%	169 ± 3	0.502 ± 0.028
D2		5%	187 ± 2	0.450 ± 0.009
D3	Trehalose	2%	206 ± 3	0.530 ± 0.027
D4		5%	212 ± 6	0.526 ± 0.019
D5	Mannose	2%	221 ± 2	0.495 ± 0.010
D6		5%	269 ± 1	0.512 ± 0.007
D7	Mannitol	2%	240 ± 11	0.562 ± 0.025
D8		5%	Unsuitable resuspension	
E1	Sucrose	2%	305 ± 9	0.259 ± 0.058
E2		5%	298 ± 5	0.187 ± 0.046
E3	Trehalose	2%	306 ± 3	0.308 ± 0.056
E4		5%	361 ± 5	0.284 ± 0.032
E5	Mannose	2%	289 ± 2	0.145 ± 0.048
E6		5%	401 ± 5	0.347 ± 0.025
E7	Mannitol	2%	412 ± 6	0.268 ± 0.042
E8		5%	Unsuitable resuspension	
F1	Sucrose	2%	550 ± 10	0.424 ± 0.085
F2		5%	332 ± 6	0.452 ± 0.015
F3	Trehalose	2%	473 ± 13	0.504 ± 0.071
F4		5%	528 ± 7	0.804 ± 0.015
F5	Mannose	2%	437 ± 38	0.728 ± 0.207
F6		5%	415 ± 7	0.472 ± 0.023
F7	Mannitol	2%	500 ± 7	0.507 ± 0.016
F8		5%	279 ± 5	0.508 ± 0.018
G1	Sucrose	2%	244 ± 17	0.731 ± 0.146
G2		5%	396 ± 28	0.751 ± 0.181
G3	Trehalose	2%	297 ± 6	0.591 ± 0.017
G4		5%	352 ± 1	0.618 ± 0.005
G5	Mannose	2%	323 ± 14	0.902 ± 0.125
G6		5%	287 ± 1	0.558 ± 0.002
G7	Mannitol	2%	278 ± 10	0.495 ± 0.032
G8		5%	236 ± 2	0.456 ± 0.004
H1	Sucrose	2%	214 ± 1	0.468 ± 0.008
H2		5%	243 ± 2	0.433 ± 0.021
H3	Trehalose	2%	230 ± 2	0.493 ± 0.027
H4		5%	264 ± 4	0.472 ± 0.023
H5	Mannose	2%	205 ± 1	0.517 ± 0.004
H6		5%	290 ± 5	0.477 ± 0.025
H7	Mannitol	2%	210 ± 2	0.505 ± 0.036
H8		5%	188 ± 2	0.405 ± 0.009
